# Supplementary material for: Temporal dependence of shifts in mu opioid receptor mobility at the cell surface after agonist binding observed by single-particle tracking
Source: Sci Rep. 2019 May 13;9:7297. doi: 10.1038/s41598-019-43657-x (PMC6514008; doi:10.1038/s41598-019-43657-x)
Supplement: Supplementary file 1 — Supplementary Information [file 41598_2019_43657_MOESM1_ESM.pdf]

Supplemental Figures

Temporal dependence of shifts in mu opioid receptor mobility at the cell surface after agonist binding observed by single-particle tracking

Marissa J. Metz[1,#], Reagan L. Pennock[1,+,#], Diego Krapf[2,3,*], Shane T. Hentges[1,*]

[1]Department of Biomedical Sciences, Colorado State University, Fort Collins, CO, USA.

[2]Department of Electrical and Computer Engineering, Colorado State University, Fort Collins, CO, USA.
[3]School of Biomedical Engineering, Colorado State University, Fort Collins, CO, USA.

[+]Current address: Department of Neurobiology, University of Alabama at Birmingham, Birmingham, AL, USA.

*Corresponding authors: S.T.H., Colorado State University, 1617 Campus Delivery, Fort Collins, CO 80521, USA. hentgess@colostate.edu D.K., Colorado State University, 1301 Campus Delivery, Fort Collins, CO 80523, USA. diego.krapf@colostate.edu

[#,*]These authors contributed equally to the work

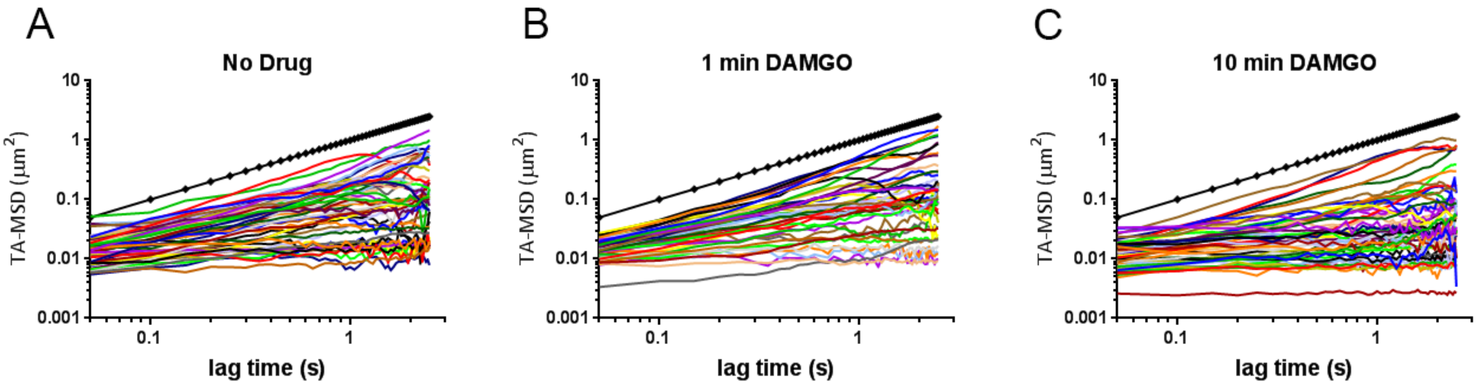

Figure S1. Individual MSDs of randomly chosen trajectories. Log-log plots of time averaged MSDs from individual tracks in the **A)** no drug, **B)** 1 min DAMGO, and **C)** 10 min DAMGO experimental conditions. Most trajectories are subdiffusive, as they lie below the slope of a simulated track of α = 1 (black dotted line).

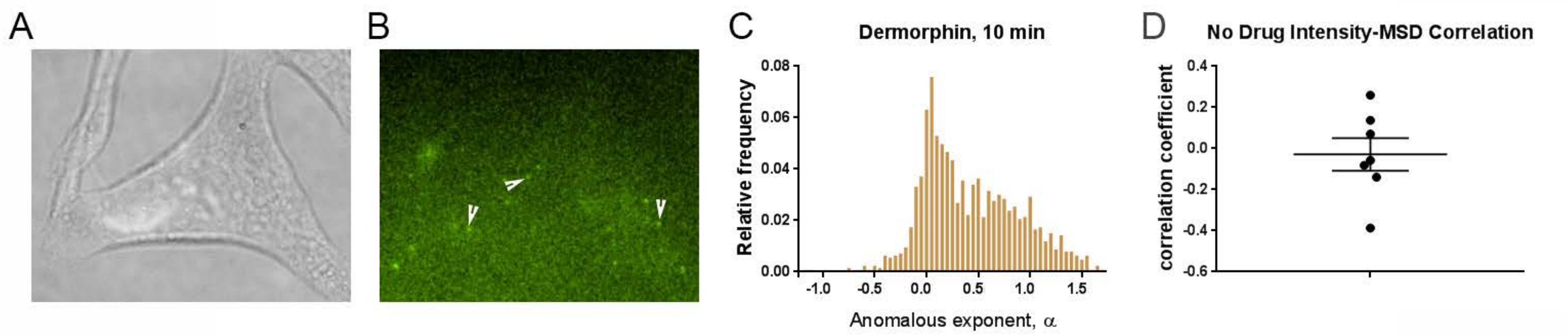

Figure S2. Single particle tracking after 10 min of Dermorphin-488 (60 pM) application reveals that the immobile population of MORs is not due to antibody-mediated crosslinking or Qdot hindering of mobility. A. DIC image of an AtT20 cell labeled with Dermorphin-488. **B)** The same cell is shown under fluorescence, and arrows indicate MORs labeled with a single Dermorphin-488 conjugate. **C)** Distribution of α values after tracking of MOR-Dermorphin-488 conjugates (n = 5 cells, 1266 tracks) incubated for 10 min in the presence of 60 pM Dermorphin-488. The distribution of α values is similar to those observed with Qdot tracking, and the fraction of $\alpha < 0.27$ is 0.44 ± 0.08, similar to MOR-Qdots in the 10 min DAMGO condition (0.45 ± 0.12). Values below 0 are likely due to errors made during tracking caused by the low signal to noise ratio with this labeling approach. **D)** Distribution of MSD vs. fluorescence intensity correlation coefficients for individual cells in the no drug condition, with a mean correlation coefficient of -0.03 ± 0.08.

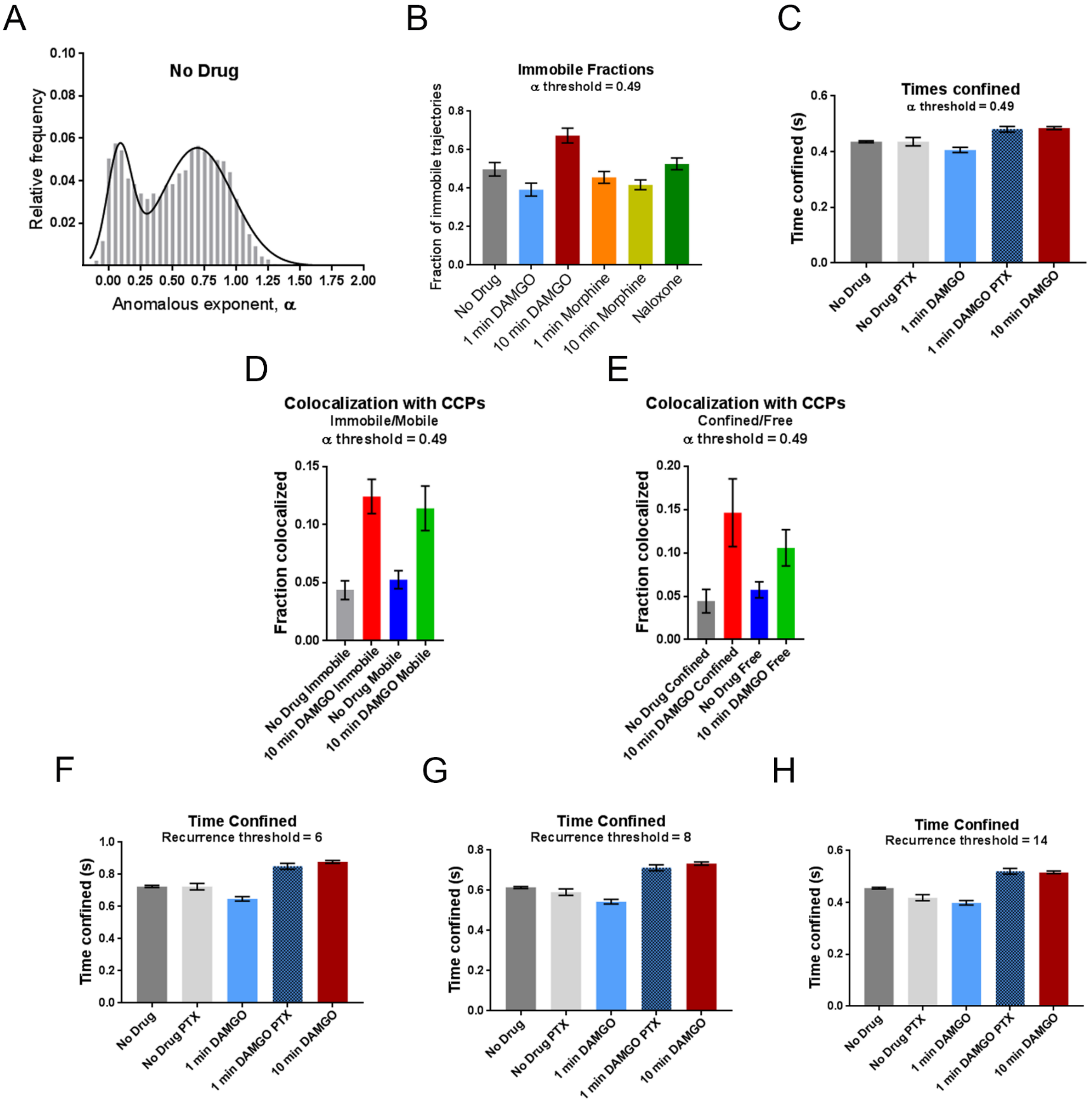

Figure S3. Reported results are robust to changes in mobile/immobile and confined/free thresholds. **A)** Two Gaussian fitting results in a threshold near our chosen threshold of α = 0.27. **B)** The pattern for fraction of immobile trajectories does not change when using the threshold of α = 0.49 determined

using k-means for most experimental conditions, with the exception of 10 min Morphine. **C)** The pattern of confined times does not change at an $\alpha = 0.49$ threshold. **D)** The pattern of colocalization with CCPs does not change when comparing immobile and mobile trajectories or **E)** confined and free trajectories. **F)** Changing the recurrence threshold during recurrence analysis does not change the time confined when the recurrence threshold is 6, **G)** 8, **H)** or 14.

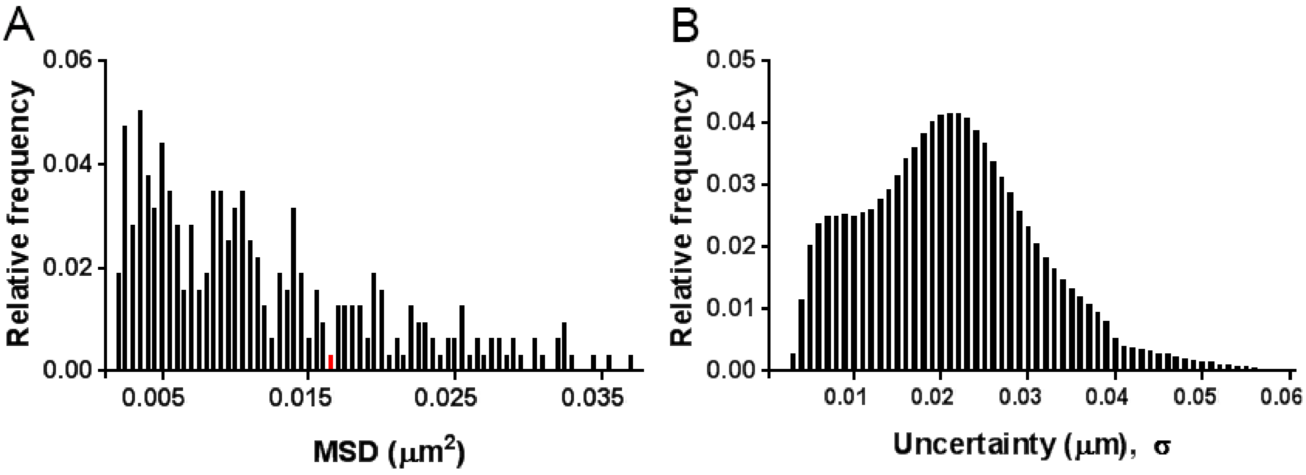

Figure S4. Data for trajectory corrections. A) Frequency histogram of MSD for glass-stuck Qdot-655 tracked without cells on the coverslip. The cutoff of 0.0165 μm^2 is highlighted in red. Most MSDs are less than this cutoff. **B)** Histogram of localization uncertainties, σ, for each detected particle of a representative subset in the no drug condition. An average σ of 0.02 μm was used for uncertainty correction of tracks.
